# Clinical Experience of Use of Percutaneous Continuous Nervemonitoring in Robotic Bilateral Axillo-Breast Thyroid Surgery

**DOI:** 10.3389/fendo.2021.817026

**Published:** 2022-02-08

**Authors:** Daqi Zhang, Cheng Wang, Tie Wang, Rui Du, Kunlin Li, Mingyu Yang, Gaofeng Xue, Gianlorenzo Dionigi, Hui Sun

**Affiliations:** ^1^Division of Thyroid Surgery, China-Japan Union Hospital of Jilin University, Jilin Provincial Key Laboratory of Surgical Translational Medicine, Changchun, China; ^2^Division of General Surgery, Istituto Auxologico Italiano, Milan, Italy; ^3^Department of Pathophysiology and Transplantation, University of Milan, Milan, Italy

**Keywords:** thyroid, surgery, nerve monitoring, percutaneous, recurrent laryngeal nerve

## Abstract

**Introduction and Objective:**

There is a need for a simplified technique for C-IONM in robotic surgery. The primary aim of this study was to describe our clinical experience with the use of percutaneous C-IONM in robotic bilateral axillary thyroid surgery.

**Methods:**

This study prospectively enrolled 304 consecutive patients who underwent robotic thyroidectomy *via* the bilateral axillo-breast approach and standardized C-IONM *via* percutaneous probe stimulation.

**Results:**

323 RLNs were analyzed. C-IONM with percutaneous probes was feasible in all cases. During this study, we did not record any cases of probe displacement, and no additional robotic maneuvers were required. The average stimulation intensity was 2 mA. There were no adverse local or systemic C-IONM side effects. The mean time required for probe positioning was 3 minutes. The EMG amplitude signal of 48 RLNs decreased significantly, < 50% from the original V1 signal. In these cases, the surgical procedure was modified.

**Conclusion:**

The proposed percutaneous C-IONM provides a simplification of the continuous monitoring procedure for robotics. The advantage of percutaneous C-IONM is that it does not require additional trocar space, repeated instrument changes, and unmodified cosmesis. To our knowledge, this is the first study on the application of percutaneous C-IONM in robotic thyroid surgery.

## Introduction

In the last 2 decades, minimally invasive thyroid surgery and intermittent intraoperative neuromonitoring (I-IONM) have been widely adopted.

During robotic thyroid surgery, transient and permanent recurrent laryngeal nerve (RLN) injuries of 3.8%-16% and 0%-0.9%, respectively, have been reported (1–5).

The use of I-IONM *via* a percutaneous probe has recently been shown to be a useful adjunct method for identifying nerves during robotic surgery. However, with I-IONM, the RLN lesion is not detected until it has already occurred (6, 7).

Continuous intraoperative neuromonitoring (C-IONM) can detect minor changes in nerve function in real time to indicate incipient RLN injury during thyroid surgery (8, 9).

Changes in technology, surgical approach, endoscope viewing direction, and dissection in robotic thyroid surgery have complicated the application of C-IONM. Therefore, there is a greater need for a simplified technique for C-IONM in minimally invasive surgery.

There have been previous studies that have attempted to simplify the application of the C-IONM (**Tables 1**, **2**).

**Table 1:** New modalities for C-IONM.

Author, year[REF]	Stimulating Route	Procedure
Zhang D, 2020 (10)	Proximal RLN	Pre-Prototype Stimulating and Recording Endotracheal Tube
Zhang D, 2019 (11)	Proximal RLN	Stimulating and dissecting instruments (endoscopy)
Zhang D, 2018 (12)	VN	Percutaneous VN stimulation
Sinclair CF, 2018 (13)	RLN	Utilizes endotracheal tube electrodes to RLN simultaneously stimulate laryngeal mucosa and record a laryngeal adductor reflex continuous IONM response
Liu XL, 2016 (14)	Proximal RLN	Continuously stimulating the RLN at the lower exposed end with a hand probe stimulator
Chiang FY, 2015 (15)	Proximal RLN	Stimulating and dissecting instruments (open surgery)
Wu CW, 2013 (16)	VN	Repose the hand probe stimulating device on the VN carotid sheath while thyroid dissection
Lamade W, 2007 (17) Ulmer C, 2008 (18) Schneider R, 2009 (19)	VN	VN cuff electrode
Lamade W, 1997 (20)	Proximal RLN	Trans-tracheal by endotracheal tube
Smith DB, 1989 (21)	RLN	Double-ballooned endotracheal tube and pressure transducer system

C-IONM, continuous nerve monitoring; RLN, recurrent laryngeal nerve; VN, vagal nerve.

**Table 2:** Classification of C-IONM modalities.

***Site of stimulation***
•VN route
•Most proximal RLN
•RLN
***Invasiveness***
•Non-invasive (i.e. trans-cutaneous/trans-tracheal/endotracheal tube based)
•Minimally invasive (V3 Inomed probe, percutaneous)
Open (Delta Inomed probe, APS Medtronic probe, Saxophone Langer Probe)
***VN degree of dissection***
•360° (Delta, APS, Saxophone probes)
•Partial (S shaped Langer probe)
No dissection (V3)
***Eletrical stimulation***
•Monopolar
•Bipolar
Tripolar
***Mode of Stimulation***
•Continuous
Periodic

V3 & Delta Inomed, Medizintechnik GmbH, Germany.

Automatic Periodic Stimulation™ (APS) Medtronic, Minneapolis, Minnesota, USA.

S shaped Dr Langer Medical, Waldkirch, Germany.

The primary aim of this study was to describe our clinical experience with the use of percutaneous continuous nerve monitoring in robotic bilateral axillary thyroid surgery.

## Materials and Methods

### Study Design

Prospective study

### Setting

Department of Thyroid Surgery, China-Japan Union Hospital, Jilin University, China

### Time Frame

Between April 3, 2020 and November 5, 2021

### Ethics

This study was approved by the Ethics Committee of the Institutional Review Board of Jilin Provincial Key Laboratory of Surgical Translational Medicine, China-Japan Union Hospital of Jilin University. Informed consent was obtained from all participants in this report.

### IONM Training of Personnel

All staff involved in the present study have 10 years of experience with I-IONM and C-IONM with more than 1,500 procedures/year.

### Patients

This study prospectively enrolled 304 consecutive patients who underwent robotic thyroidectomy *via* the bilateral axillo-breast approach and standardized C-IONM *via* percutaneous probe stimulation by the same surgeon (D.-Q.Z.).

### Inclusion and Exclusion Criteria

**Table 3** summarizes the selection criteria for robotic thyroidectomy.

**Table 3:** Detailed inclusion and exclusion criteria for BABA.

Selection and exclusion criteria
Selection criteria
Papillary thyroid cancer with low-risk factors ^a^
Dominant benign nodule with a diameter <5 cm, whereas cystic nodule could be 6 cm or greater
The patients needed a cosmetic requirement
Exclusion criteria
General factors
Obesity
Clinical history of radiation or surgery on the neck or chest
Preoperative dysfunction of voice cord
Thyroid-related factors
Advanced cancer
Local invasion
Posteriorly located lesions
Diffuse or adhesion or fixation enlargement of lymph node
Evidence available of local or distant metastases
Graves’ disease
Severe thyroiditis
Associated parathyroid disease

a Low risk factors including lesion size <4 cm, age <55 years, no prior radiation, no distant metastases, no lymph node metastases, no extrathyroidal extension, no aggressive variant, and no first-degree family history of thyroid carcinoma.

### Percutaneous Nerve Stimulation

#### Neural Monitoring System

The IONM system used in this study (NIM -Response 3.0 System; Medtronic, Jacksonville, FL) includes multiple recording, stimulation, and connection devices that display complete EMG waveforms, parameter metrics, and audible cues while monitoring the nerve. The pulse stimuli had a magnitude of 100 l and were repeated at 4 Hz. The response threshold was set at 100 lV. Endotracheal tube-based surface electrode systems were used (tube size 6.0 inner diameter for women and 7.0 for men, standard electromyography (EMG) tube (Medtronic). The correct depth and position of the EMG tube electrodes was verified by laryngoscopy.

#### Stimulating Probe

**Figure 1:**
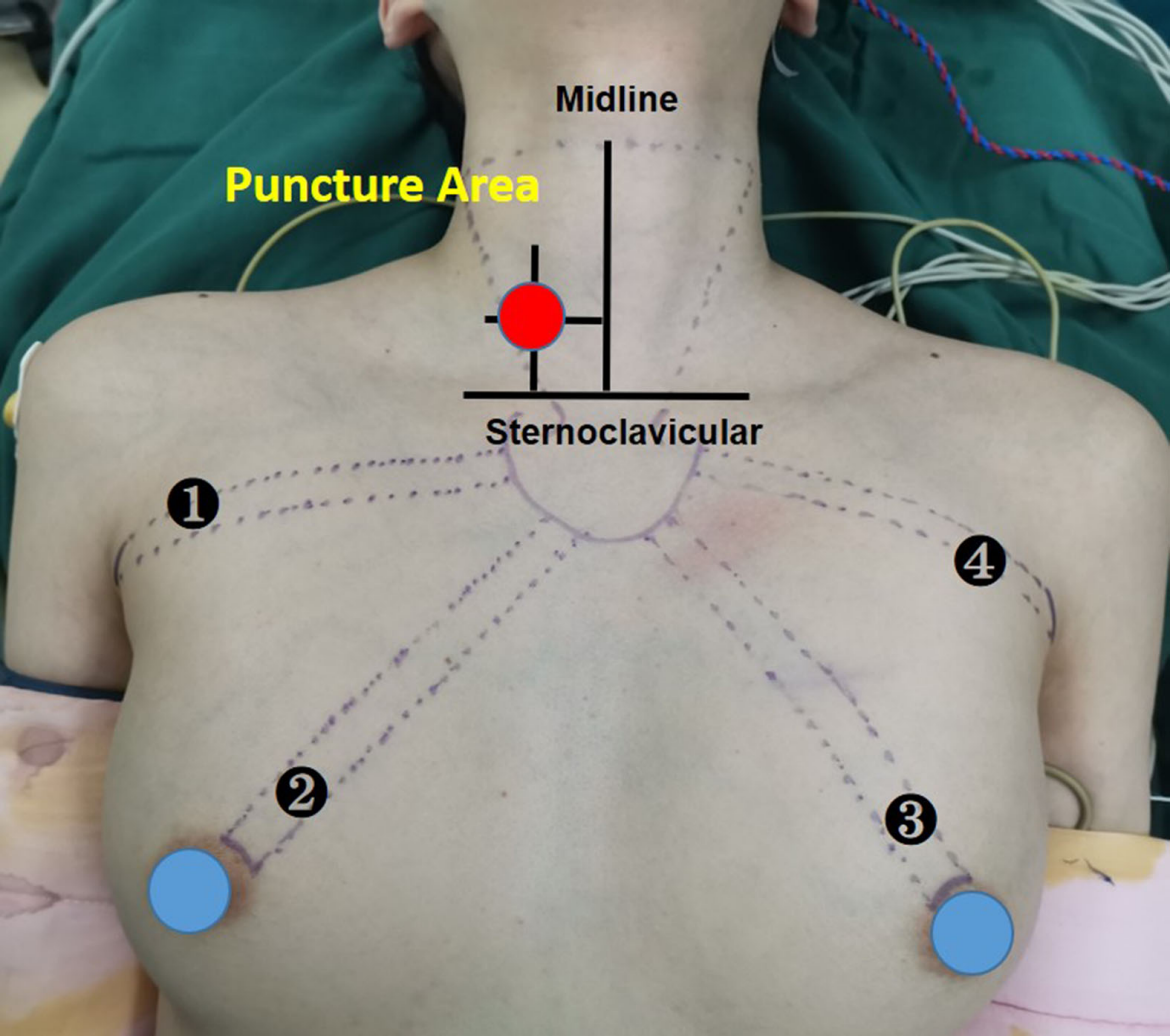
Selection of puncture point for nerve-monitoring probe.

**Figure 2:**
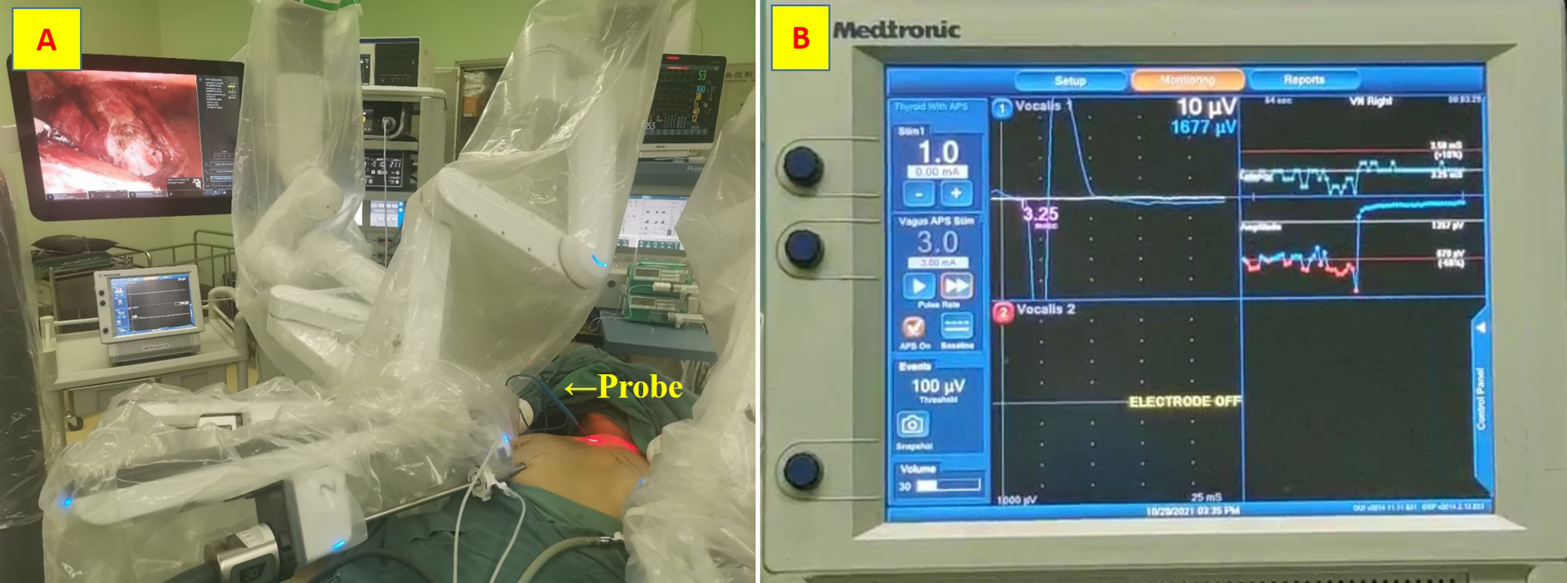
Stimulating probe. **(A)** the probe was carefully inserted through the needle channel; **(B)** Continuous neuromonitoring in APS mode.

A disposable monopolar ball-tipped stimulating probe (1.0 mm) (product number PSP1002, Medtronic) with a 10-cm handle and a 9-cm shaft was used for nerve stimulation through a percutaneous approach. A 0.5-cm circle was drawn on the side of the dominant thyroid lesion, with its midpoint at the intersection of a line 2 cm lateral to the anterior median line and a line 2 cm above the line connecting the bilateral clavicular heads (12) (**Figure 1**). After ensuring that there were no major vessels within the puncture site in this circle, the skin was pierced with an 18-gage hypodermic needle. After the needle was withdrawn, the probe was carefully inserted through the needle channel (**Figure 2A**). The tool is usually guided by the first assistant. During dissection from the junction of the inferior thyroid artery and the recurrent laryngeal nerve to the larynx, the stimulator tip is gently held on the vagus nerve to allow continuous neuromonitoring in APS mode. (**Figure 2B**).

#### Definitions

The IONM standardized technique includes the determination of L1, V1, R1, C1, C2, R2, V2, and L2. V1 is stimulation of the vagus nerve (VN) prior to surgical dissection. R1 is the stimulation of the RLN at initial identification. C1 is the initial value for continuous monitoring. C2 is the final value for continuous monitoring. V2 and R2 are the stimulations of the VN and RLN, respectively, after completion of thyroid dissection. In all patients, vocal cord motion was assessed by laryngofiberoscopy before surgery (L1) and on the morning of the first day after surgery (L2). Any restriction of vocal cord movement was recorded as postoperative vocal cord paralysis. RLN paralysis was defined as permanent if there was no evidence of recovery within 6 months after surgery. The definition of decreased EMG amplitude or loss of signal (LOS), the types of RLN injury (type 1 segmental and type 2 global), the mechanism of injury, and the troubleshooting algorithm were taken from published guidelines (22).

**Obesity** According to the suggestions of the WHO for Chinese populations, obesity was defined as BMI ≥ 27.5 kg/m2 (23).

**Thyroiditis severity** was characterized according to Effraimidis and Feldt-Rasmussen definition (24).

### C-IONM Procedure

In this Institution, robotic thyroidectomy is performed *via* a bilateral axillo-breast approach. The steps of the continuous monitoring mode of the percutaneous probe are as follows (**Video 1**):

(A)Incisions and port insertion: the right nipple incision was placed in the 30-degree laparoscope, and the left nipple incision, left axillary incision, and right axillary incision were placed using an energy-based device, Maryland dissection forceps, and ProGrasp forceps, respectively.
(B) Creation of the working space: the superior border is represented by the superior border of the thyroid cartilage, laterally by the medial border of the sternocleidomastoid muscle, inferiorly by the sternum, posteriorly by the strap muscles.
(C) Insertion of the probe as described above.
(D) Vagus nerve stimulation: to check the integrity of vagus nerve function, the probe was pushed with the ball tip into the space between the common carotid artery and the internal jugular vein to obtain V1 under stimulation set at 3 mA.
(E) Mapping and identification of the RLN (R1): Mapping is achieved with stimulation of 2 to 3 mA at the inferior pole of the thyroid gland. The RLN was tested with a stimulation of 1 mA after visualization of the RLN but before dissection, and the EMG signal was recorded as R1.
(F) Dissection of the thyroid gland with intermittent nerve monitoring: Inferior thyroid vessels were then coagulated with the ultrasound scalpel, and the thyroid lobe was dissected in the caudal to cranial direction while the endoscope was used for a clear view of the course of the RLN. The RLN was stimulated at least once per minute to monitor EMG amplitude until the junction of the inferior thyroid artery and recurrent laryngeal nerve was exposed.
(G) Thyroid dissection with continuous nerve monitoring:During the dissection from the junction of the inferior thyroid artery and the recurrent laryngeal nerve to the larynx, the ball tip of the probe was pushed into the space between the common carotid artery and the internal jugular vein in APS mode. Then, the standard 20 pacing pulses and measurements were performed to establish baseline and record C1 and C2. Once LOS occurs, surgical maneuvers were modified or paused for a maximum of 30 minutes.
(H) Final check of the functional integrity of the recurrent laryngeal nerve: After thyroidectomy and hemostasis, the most proximal exposed points of the RLN (R2) and vagus nerve (V2) were retested to assess RLN function.

### Sample Size Calculation

For calculation of sample size, we assumed a baseline risk of C-IONM failure/probe displacement/complications (based on literature search) as 5-10% in robotic thyroidectomy (25). We hypothesized that to detect such a difference with 5% margin of error and 95% confidence, we would need a minimum sample of 300 procedures.

### Statistical Evaluation

We used SPSS version 23 (IBM Inc., USA) for statistical analysis. Continuous variables are presented either as mean ± standard deviation (SD). A P value of less than 0.05 was considered significant.

## Results

### Demographic and Procedural Characteristics of Patients Enrolled in the Study

All 304 patients successfully completed robotic thyroidectomy, and no patient was referred for open surgery. The mean age was 33 years, male/female ratio was 29/275, mean BMI was 22.3, and most of them were normal or underweight (221 cases, 72.7%). Postoperative pathological results showed that 41 cases were benign tumors, 263 cases were malignant tumors, and 33 cases were complicated thyroiditis. The median operative time was 140 minutes. The median postoperative drainage volume was 52.5 ml, and the median postoperative hospital stay was 3 days. IONM showed that 48 patients had electromyographic changes, and 10 patients were diagnosed with transient recurrent laryngeal nerve palsy by postoperative laryngoscopy. Intraoperative parathyroid autotransplantation was performed in 6 patients, and transient hypocalcemia occurred in 2 patients postoperatively (**Table 4**).

**Table 4:** Clinicopathological data of 304 patients with thyroid surgery.

Characteristics	n (%)
**Age (year)**	33(27;41)
<45	260(85.5)
45-55	44(14.5)
**Gender**	
male	29 (9.5)
female	275(90.5)
**BMI**	22.3(20.5, 25.4)
normal (<25)	221(72.7)
fat (≥25)	83(27.3)
**Pathology**	
benign	41(13.5)
malignant	263(86.5)
**Maximum tumor (cm)**	
benign	3.5(3.0;4.0)
malignant	0.6(0.4, 0.9)
**With thyroiditis**	
yes	33(14.1)
no	271(85.9)
**Surgery duration (min)**	140(121, 170)
**Postoperative drainage (ml/d)**	65(50, 80)
**Postoperative hospital stay (day)**	3(2, 7)
**RLN**	
Total	323
EMG change	48
Temporary vocal cord paralysis	10
Permanent vocal cord paralysis	0
**Parathyroid**	
Temporary hypocalcemia	2
Permanent hypocalcemia	0
Parathyroid transplantation	6

### Clinical and C-IONM Outcomes

323 RLNs were analyzed. Continuous monitoring mode with percutaneous probes was feasible in all cases. During this study we did not record any cases of probe displacement, no additional robotic maneuvers were required. The mean stimulation intensity used was 2mA. There were no C-IONM adverse local or systemic side effects. Mean time effort for probe positioning was 3 min. **Table 5** shows that the EMG amplitude signal of 48 RLNs decreased significantly, < 50% from the original V1 signal. In these cases, the surgical procedure was immediately stopped for 30 minutes and the correct position of the EMG endotracheal tube electrode was confirmed with the laryngofiberscope. Proximal and distal mapping of the RLN course revealed that 43 RLNs had segmental injuries with weaknesses in the inferior thyroid artery (ITA) or Berry’s ligament. An additional 5 RLNs had a global injury with no evidence of an interruption site on the exposed nerve. Forty-five nerve injuries resulted from compression by the ITA or compression by fibrotic bands of Berry’s ligament during retraction and dissection of the thyroid lobe. 3 Nerve injuries resulted from thermal injury from the ultrasonic knife. After 30 minutes of surgical standby, 38 cases recovered to > 50% R1 with normal vocal cord movement on the first postoperative day. Another 10 cases who recovered to < 50% R1 showed abnormal vocal cord movement on the first postoperative day. Of these, there were five cases whose EMG signals remained stable before wound closure. All vocal cord fixations were recovered 2-5 months postoperatively. No permanent vocal cord paralysis occurred in any of the cases.

**Table 5:** EMG changes of RLN and vocal cord dysfunction in RLN.

	No. of RLNs
**EMG loss maximal >50%**	**48**
**Type of injury**	
**Segmental**	**43**
**Global**	**5**
**Injury Mechanism**	
**Compression**	**45**
**Vascular heat conduction**	**3**
**Recovery performances of EMG**	
**Recovered to an amplitude of >50% V1**	**38**
**Recovered to an amplitude of <50% V1**	**5**
**Never recovered**	**5**
**Vocal cord dysfunction**	**10**
**Transient/Persistent**	**10/0**

## Discussion

Robotic-assisted surgery is becoming increasingly popular among surgeons because it offers a high-resolution field of view with 10-15x magnification, a robotic arm with greater freedom, and tremor filtering. The proliferation of robotic thyroid surgery also brings new challenges to the application of C-IONM in robotic thyroid surgery. With conventional stimulation probes, it is difficult to monitor the nerve on the external cervical route (10–19). The proposed percutaneous C-IONM provides a simple, cost-effective, and efficient solution to this challenge. Placement of the probe within the above area allows both avoidance of the anterior cervical vessels and monitoring of the vagus nerve and RLN in the resected lateral cervical segment. The percutaneous C-IONM probe is out of the surgical maneuvering space and not interfering endoscopic visualization or other instruments. The other advantage of percutaneous C-IONM is that it does not require an additional trocar space and does not require repeated instrument changes. The procedure is cosmetic and leaves only a 1-mm needle hole in the neck, which usually heals completely within 1 day after the procedure. In the literature there are technical reports on the use of C-IONM in robotic thyroid surgery (26) as well as experimental and original studies with cases series mainly for the transaxillary and vestibular approach (27). We reported the first successful application of C-IONM during trans-areola thyroidectomy in a large series of patients. Regarding the technological requirements, no additional electrodes are needed to stimulate the vagus nerve and related supportive equipment, and no additional opening of the cervical sheet is necessary.

In this study, percutaneous C-IONM was performed in 304 patients and 323 RLNs. 48 RLNs showed a 50% decrease in EMG signal intraoperatively. The change in surgical manoeuvre resulted in varying degrees of EMG recovery. Transient vocal cord paralysis was observed in 10 patients postoperatively. The rate of transient vocal cord paralysis was 3.1%, and all returned to normal 2-5 months after surgery. No other local or systemic adverse side effects were observed during use.

The mechanism and site of nerve damage were also elucidated. The area of the RLN and the ligamentum Berry in the part of the inferior thyroid artery that crosses the entry site into the larynx are the most vulnerable sites for injury. Traction injuries and energy-related thermal injuries were the most common causes of injury. This is consistent with other studies (28–33).

The C-IONM allows the modification of surgical maneuvers in combination and simultaneously with the reduction of EMG signal amplitude and increase of latency. This is not the goal of this work, but the settings most commonly used by the surgeon in response to nerve stress feedback are: (a) release of traction of the thyroid lobe, (b) avoidance of the use of EBD near the nerve, (c) surgical on-call avoiding any nerve transection, (d) use of cortisone (iv or topically in the surgical area).

In our previous experience, we routinely used an intermittent monitoring mode in both endoscopy and open surgery, in which the RLN was periodically stimulated to monitor quantitative changes in EMG amplitude during detachment of the thyroid gland. This showed postoperative vocal cord palsy rates of 7.0% for endoscopy and 4.0% for open surgery (12, 31). Compared with the intermittent monitoring mode, the continuous monitoring mode we used in robotic thyroid surgery allows uninterrupted real-time monitoring of RLN function through continuous stimulation of the vagus nerve, and the surgeon can understand and stop potentially nerve-inducing surgical procedures in a timely manner based on changes in EMG, which contributes to the recovery of RLN function after transient LOS.

A limitation of this study is that this was a prospective cohort observational study in which all participants underwent the same protocol. We did not randomize any group. The study design did not include a control group that could be used to demonstrate cause and effect with some degree of certainty. Certainly, a randomized trial with or without IONM is not feasible (because of surgeon and patient refusal to be randomized). On the contrary, in the future we will explore the possibility of using a control group offered a different I-IONM or C-IONM method.

A further limitation of the study is given by our choice to use the 5-10% C-IONM displacement as sample size. This choice was dictated by the impossibility of using other outcomes for the calculation of the sample size because they are burdened by a large number of patients to be recruited. For example, to have sufficient statistical calculation for recurrent paralysis, we need> 5,000 nerves at risk (34). This number is not currently available.

## Data Availability Statement

The original contributions presented in the study are included in the article/**Supplementary Material**. Further inquiries can be directed to the corresponding author.

## Ethics Statement

The studies involving human participants were reviewed and approved by Jilin Provincial Key Laboratory of Surgical Translational Medicine. The patients/participants provided their written informed consent to participate in this study. Written informed consent was obtained from the individual(s) for the publication of any potentially identifiable images or data included in this article.

## Author Contributions

Conception and design: HS and GD. Administrative support: HS. Collection and assembly of data: HS, GD, and DZ. Data analysis and interpretation: HS, GD, and DZ. Manuscript writing: All authors. All authors contributed to the article and approved the submitted version.

## Funding

This work was supported by the China Postdoctoral Science Foundation (no. 2017M611313), Department of Science and Technology of Jilin Province (no.20190201225JC) and Department of Finance of Jilin Province (no. 20201231SY058 and 2019SCZ028), China.

## Conflict of Interest

The authors declare that the research was conducted in the absence of any commercial or financial relationships that could be construed as a potential conflict of interest.

## Publisher’s Note

All claims expressed in this article are solely those of the authors and do not necessarily represent those of their affiliated organizations, or those of the publisher, the editors and the reviewers. Any product that may be evaluated in this article, or claim that may be made by its manufacturer, is not guaranteed or endorsed by the publisher.
